# Positive response to Icotinib in metastatic lung adenocarcinoma with acquiring EGFR Leu792H mutation after AZD9291 treatment: a case report

**DOI:** 10.1186/s12885-019-5352-7

**Published:** 2019-02-08

**Authors:** Junhui Wang, Jianxin Chen

**Affiliations:** 1 Department of Radiation Oncology, Quzhou People’s Hospital, Quzhou, 324000 Zhejiang China; 2 Department of Medical Oncology, Quzhou People’s Hospital, No. 2 Zhongloudi Road, Quzhou, 324000 People’s Republic of China

**Keywords:** EGFR, Tyrosine kinase inhibitors, Leu792H mutation, Icotinib

## Abstract

**Background:**

Epidermal growth factor receptor-tyrosine kinase inhibitors (EGFR-TKIs) have been emerged as the standard selection in non-small cell lung cancer (NSCLC) patients with EGFR sensitive mutations. However, primary or acquired resistance to EGFR-TKIs seems inevitable, especially to third-generation TKIs, which has appeared absence of effective solutions so far.

**Case presentation:**

Here we reported a NSCLC patient with EGFR sensitive mutation of deletion within EGFR exon 19, who had been resistant to icotinib and AZD9291 successively after a period of 18 months response duration. Next-generation sequencing (NGS) technique using plasma sample suggested an acquired EGFR Leu792H mutation, rather than C797S one. Interestingly, the patient obtained another 8 months of disease-free duration with symptoms greatly relieved after repeating icotinib administration. The overall survival of the patient has been thirty-six months and still in the extension.

**Conclusion:**

The presentation of the case may provide some selective therapeutic thoughts for NSCLC patients with acquired EGFR Leu792H mutation suffering resistance to the third-generation TKIs.

## Background

Owing to the well-established beneficial outcomes of clinical trials, epidermal growth factor receptor-tyrosine kinase inhibitors (EGFR-TKIs), have been approved as the standard regimens in patients with non-small cell lung cancer (NSCLC) harboring EGFR sensitive mutations [1]. However, acquired resistance to EGFR-TKIs has greatly limited their clinical administration, with few solutions detected to overcome the challenging problem. Here, we report a NSCLC patient with brain metastasis, accompanied with EGFR exon 19 deletion, as well as an emerged EGFR Leu792H mutation after the management of AZD9291, who responded positively to the repeating treatment of icotinib, has obtained a 36 months survival during so far.

## Case presentation

**Fig. 1:**
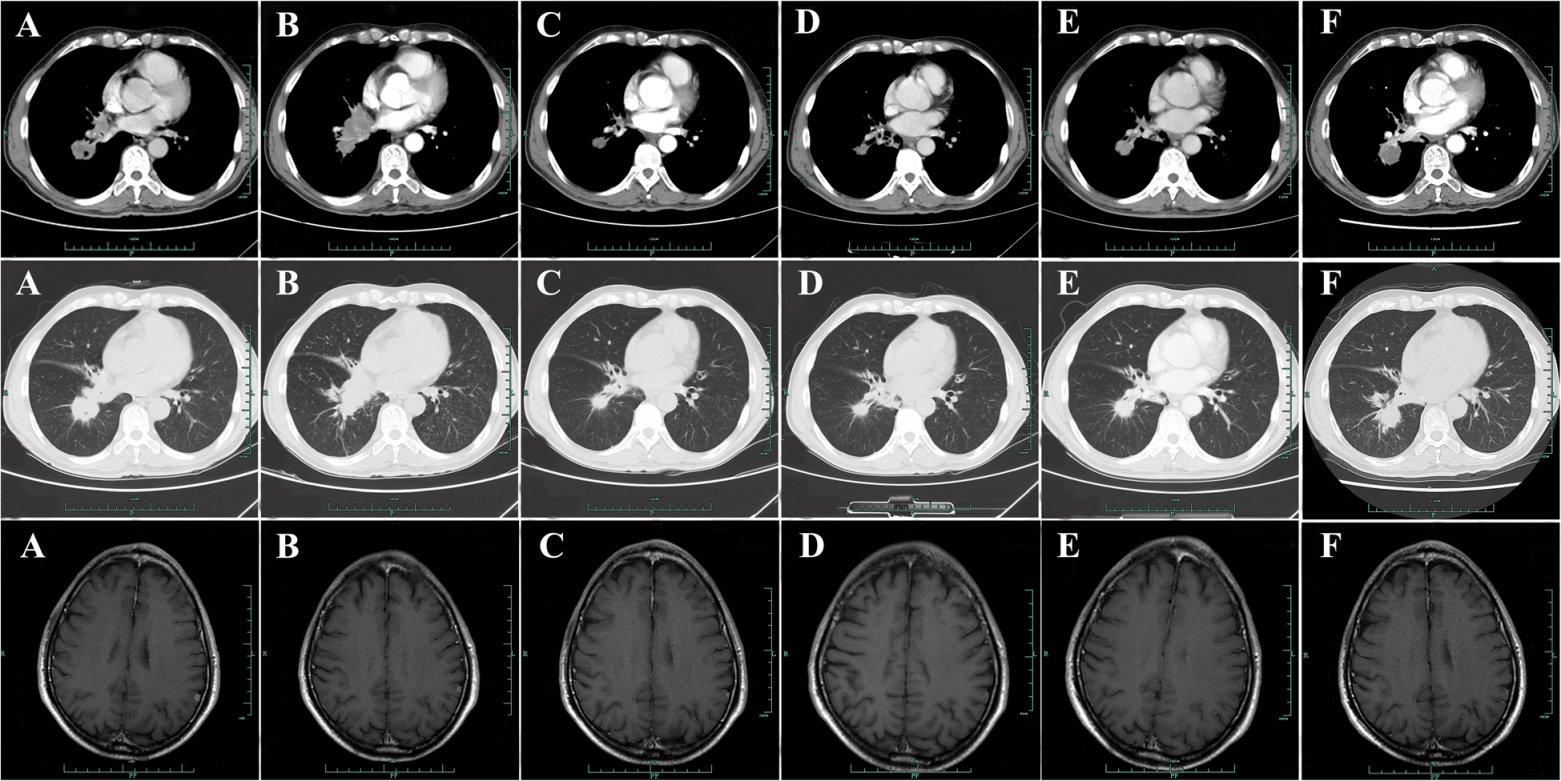
Chest CT scan and brain MRI from April 2015 to February 2016. **a** Chest CT scan and brain MRI at the time of diagnosis in April 2015. **b** Chest CT scan and brain MRI after 2 cycles of pemetrexed plus cisplatin treatment in June 2015. **c** Chest CT scan and brain MRI after 1 month treatment of icotinib in July 2015. **d** Chest CT scan and brain MRI after 3 months treatment of icotinib in September 2015. **e** Chest CT scan and brain MRI after 6 months treatment of icotinib in December 2015. **f** Chest CT scan and brain MRI after 8 months treatment of icotinib in February 2016

**Fig. 2:**
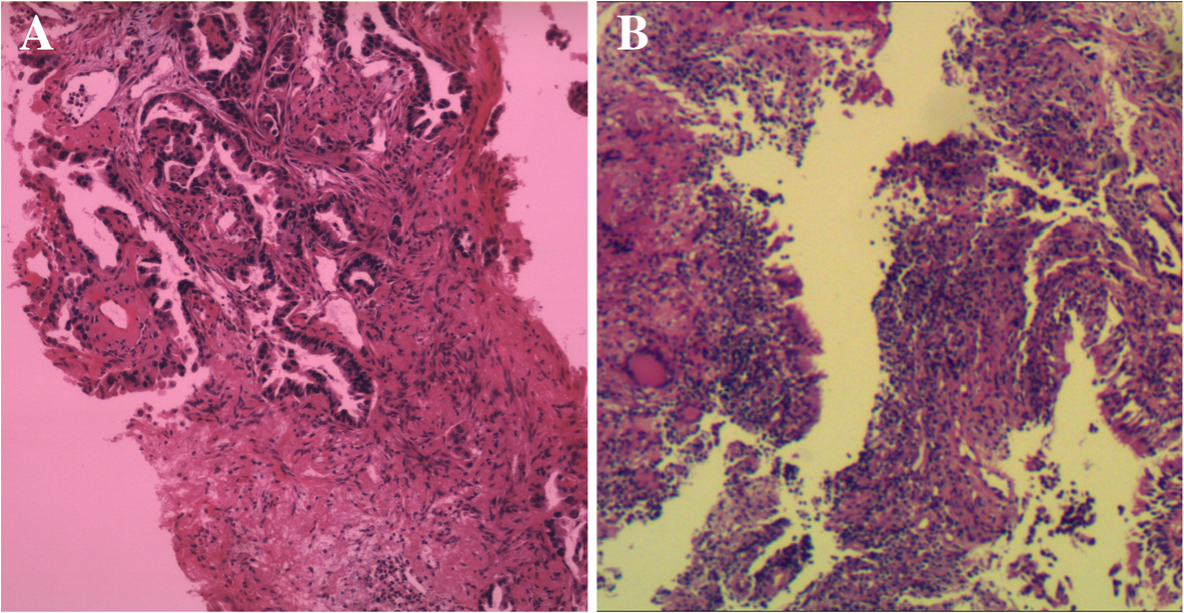
Histological findings of tumor tissue. **a** Histological finding with hematoxylin and eosin–stained biopsy specimen from PNLB on April 15th 2015. **b** Histological finding with hematoxylin and eosin–stained biopsy specimen from PNLB on December 7th 2015

**Fig. 3:**
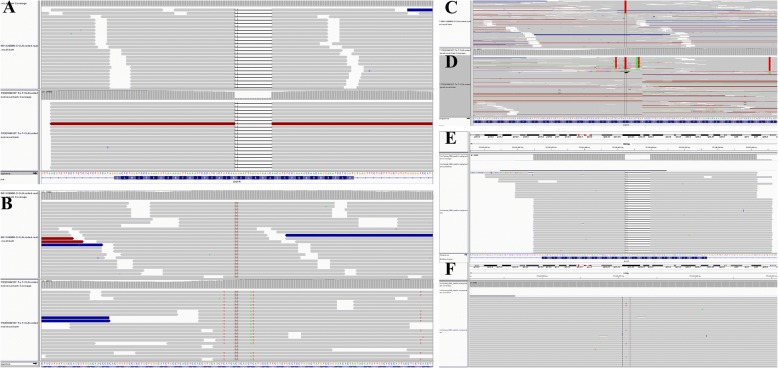
Drive gene detecting during the treatment. **a** NGS analysis of EGFR exon 19 deletion mutation in February 2016. **b** Acquiring T790 M mutation with plasma after resistant to icotinib in February 2016. **c** NGS analysis of EGFR exon 19 deletion mutation in March 2017. **d** NGS analysis of EGFR T790 M mutation, as well as Leu792H mutation with plasma after resistant to AZD9291 in March 2017. **e** NGS analysis of EGFR exon 19 deletion mutation in December 2017. **f** NGS analysis of EGFR T790 M mutation, without Leu792H mutation any more with plasma after resistant to repeating icotinib in December 2017

**Fig. 4:**
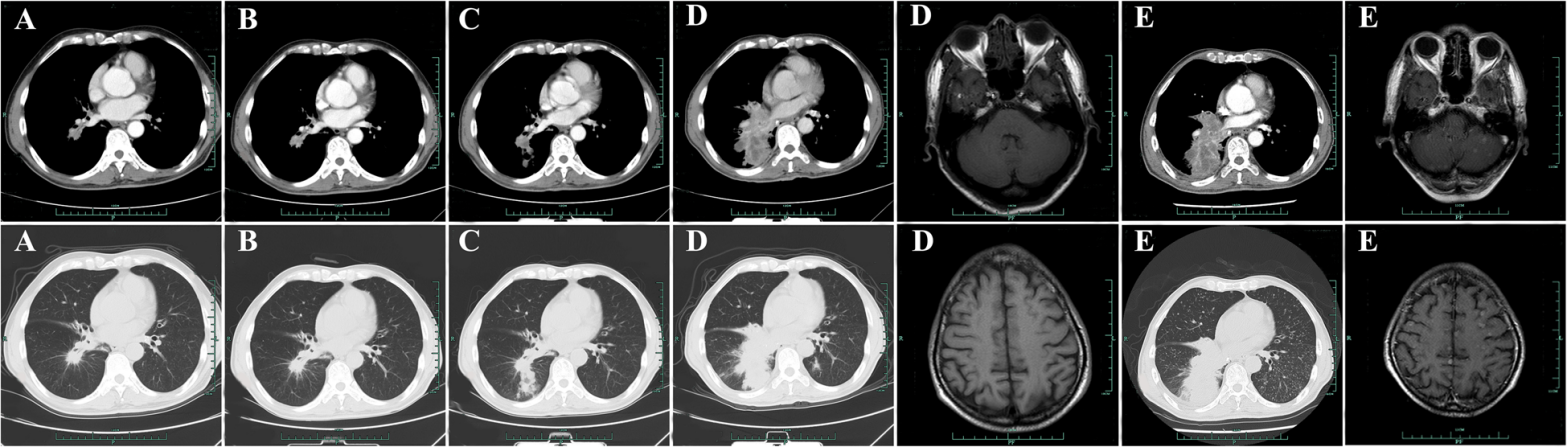
Chest CT scan and brain MRI from April 2016 to March 2017. **a** Chest CT scan after 1 month treatment of AZD9291 in April 2016. **b** Chest CT scan after 3 months treatment of AZD9291 in June 2016. **c** Chest CT scan after 6 months treatment of AZD9291 in September 2016. **d** Chest CT scan and brain MRI after 10 months treatment of AZD9291 in January 2017. **e** Chest CT scan and brain MRI after 2 cycles of docetaxel treatment in March 2017

**Fig. 5:**
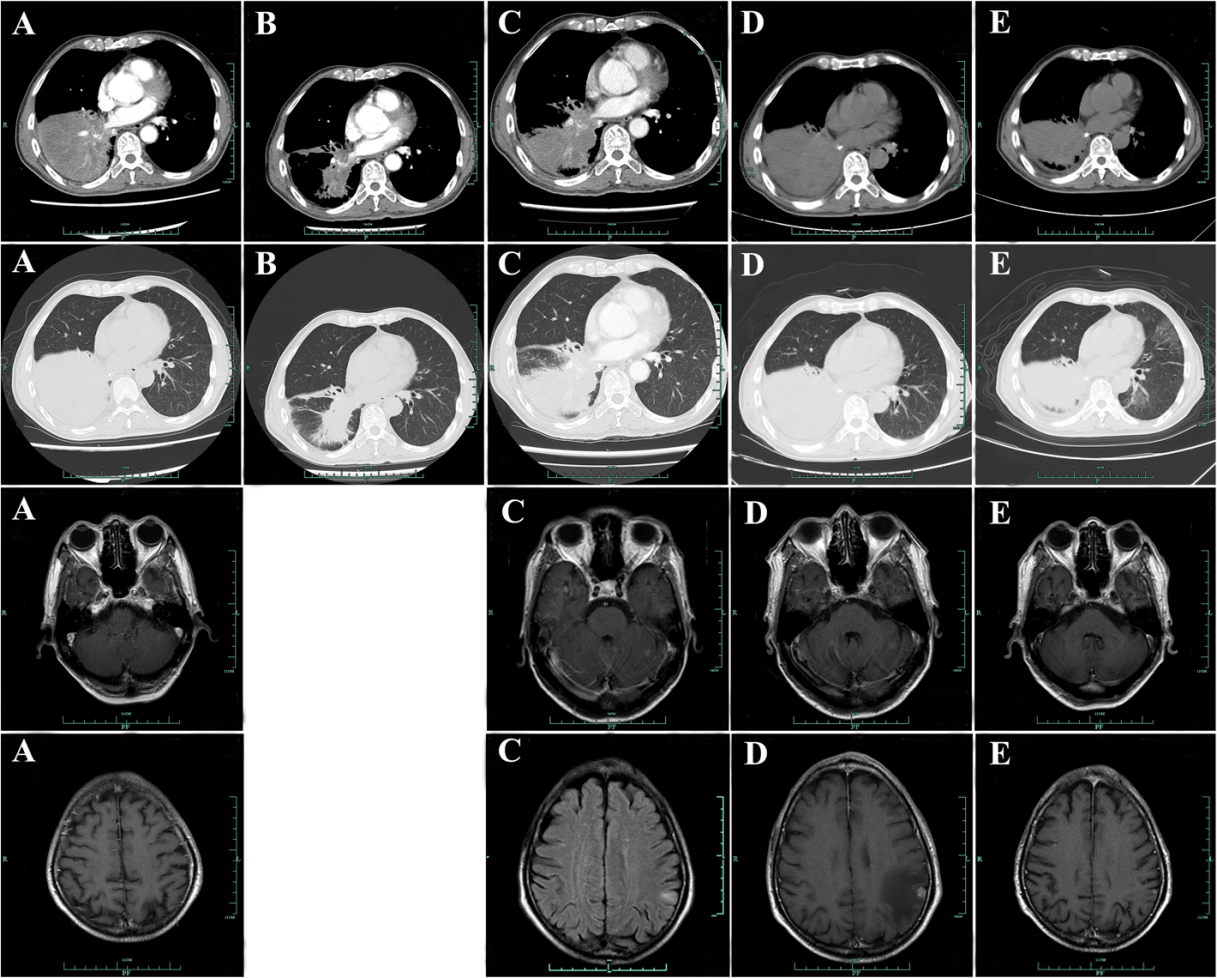
Chest CT scan and brain MRI from May 2017 to February 2018. **a** Chest CT scan and brain MRI after 2 months treatment of repeating icotinib in May 2017. **b** Chest CT scan after 3 months treatment of repeating icotinib in June 2017. **c** Chest CT scan and brain MRI after 6 months treatment of repeating icotinib in September 2017. **d** Chest CT scan and brain MRI after 8 months treatment of repeating icotinib in November 2017. **e** Chest CT scan and brain MRI after 1 month treatment of repeating AZD9291 in February 2018

**Fig. 6:**
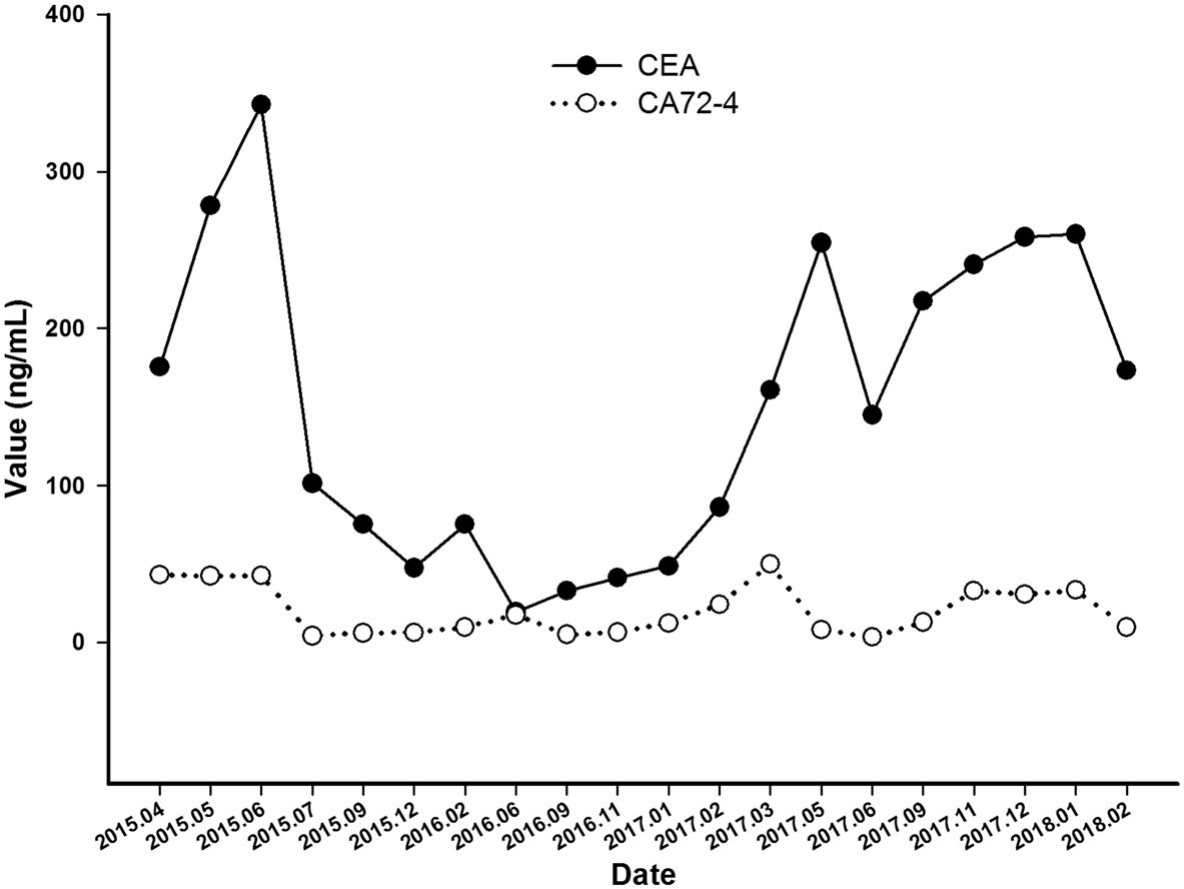
The variation CEA and CA72–4 for each visit from the initial treatment to the present

A 65-year old male patient was referred to our hospital with several space-occupying lesions in inferior lobe of right lung and enlarged lymph nodes in mediastinum and bilateral hilum detected occasionally by chest computed tomography (CT) scanning during the annual health examination in April 2015 (Fig. 1a). Sequential brain magnetic resonance imaging (MRI) showed a space-occupying lesion in left parietal lobe (Fig. 1a). One of the lesions in right lung obtained by percutaneous lung biopsy (PNLB) was proved to be adenocarcinoma (Fig. 2a). Subsequent drive gene analysis with the method of amplification refractory mutation system (ARMS) using a collected tissue sample suggested a deletion of EGFR exon 19 without T790 M mutation. Hence, a clinical diagnosis was made as adenocarcinoma in inferior lobe of right lung, in association with multiple lesions among bilateral lungs, enlargement of lymph nodes in mediastinum and bilateral hilum, and solitary lesion in left parietal lobe of brain suggesting metastasis (Fig. 1a). The patient was treated with two cycles of chemotherapy (pemetrexed plus cisplatin) as first-line therapy until June 2015, due to the delayed report of drive gene analysis. After the finish of chemotherapy, repeating chest CT scan revealed an increased lump in inferior lobe of right lung, as well as lymph nodes in mediastinum and bilateral hilum (Fig. 1b). Brain MRI in the same week showed a stable node (Fig. 1b). We therefore, evaluated the efficacy of the first-line chemotherapy as progressive disease (PD) according to response evaluation criteria in solid tumors (RECIST version 1.1). Icotinib was then administrated as the second-line treatment in June 2015. Subsequent CT scans, as expected, revealed a partial response (PR) for lesions in lung and a complete response (CR) in brain in the following 8months (Fig. 1c-e). In February 2016, regular chest CT scanning showed a secondary enlargement of neoplasm in primary location of right lung (Fig. 1f). Next-generation sequencing (NGS) technique using the patient’s plasma sample suggested an acquired T790 M mutation by frequency of 4% accompanied with the deletion of EGFR exon 19 by 7% (p.745–750 del. c.2235_2249 del GGAATTAAGAGAAGC. Figure 3a-b). The third-generation TKI of AZD9291, therefore, was prescribed as the third-line therapy in March 2016. After a ten-months duration of response (Fig. 4a-c), significant PD of lump in inferior lobe of right lung with atelectasis was detected again by chest CT scanning in January 2017 (Fig. 4d), while without any lesions in brain (Fig. 4d). Hence, two cycles of cytotoxic drug with docetaxel were administrated as the fourth-line management then. However, the re-enlargement of primary neoplasm in lung and multiply emerging lesions in brain signified a PD again in March 2017 (Fig. 4e), with symptoms of cough and hemoptysis aggravated seriously. Repeating NGS with plasma was developed on March 10th 2017. It was detected that the deletion of EGFR exon 19 (p745–750 del) with frequency by 78.3% and T790 M mutation by 0.2%, with concurring Leu792H mutation by 0.2%, rather than C797S mutation (Fig. 3c-d). In addition to those, other drive genes in the NGS panel including anaplastic lymphoma kinase (ALK), ROS proto-oncogene 1 (ROS1), V-Ki-ras2 Kirsten rat sarcoma viral oncogene homolog (KRAS), neuroblastoma RAS viral oncogene homolog (NRAS), RET proto-oncogene (RET), V-raf murine sarcoma viral oncogene homologB1 (BRAF), receptor tyrosine-protein kinase erbB-2 (ERBB2), RAC-alpha serine/threonine-protein kinase (AKT1), discoidin domain receptor tyrosine kinase 2 (DDR2), fibroblast growth factor receptor 1 (FGFR1), MNNG HOS transforming gene (MET), phosphatase and tensin homolog (PTEN), phosphatidylinosito-4,5-bisphosphate 3-kinase (PIK3CA), and mitogen-activated protein kinase 1 (MAP2K1) were detected as wild type. In view of the primary resistance to chemotherapy, lack of potentially effective selection, and high frequency of EGFR exon 19 mutation, repeating icotinib was attempted as salvage treatment. After 2 months treatment, multiply lesions in brain were dramatically disappeared according to brain MRI (Fig. 5a), as well as symptoms of cough and hemoptysis relieved apparently. The repeating icotinib was administrated till November 30th 2017 (Fig. 5b-c), on which a sudden syncope happened. An emergency brain MRI suggested recurrence of tumors, with an enlarged lesion in left parietal lobe surrounded by encephaledema severely(Fig. 5d). Coinstantaneous chest CT scanning suggested a PD of the target lesion in right lung (Fig. 5d). After symptomatic treatment of dehydration with mannitol, whole brain radiotherapy (WBRT) was developed as palliative management. PNLB was operated again on December 18th 2017, result of which reconfirm adenocarcinoma in lung (Fig. 2b), accompanied with the deletion of EGFR exon 19 (p.745–750 del) with frequency by 87.5% and T790 M mutation by 9.4%, without Leu792H mutation any more obtained from plasma or tissue (Fig. 3e-f). Sixth-line treatment of repeating AZD9291 was restarted on January 6th 2018 due to the increased frequency of T790 M mutation. One month later on February 5th 2018, repeating pictures of chest CT showed a response of lump in right lung, with partial remission of lesions in brain as MRI presented (Fig. 5e). The variation of tumor markers including carcino-embryonic antigen (CEA, normal range, 0–0.5 ng/mL) and carbohydrate antigen 72–4 (CA72–4, normal range, 0–6.9 U/mL) are showed in Fig. 6 for each visit from the initial treatment to the present. In addition, variations of tumor size during the whole treatment according to RECIST version 1.1 were listed in Table 1. The patient feels good without any symptoms and still receives AZD9291 treatment now. The overall survival has been 36 months and still in the extension.

**Table 1:** Variations of tumor size during treatment according to RECIST version 1.1

Date	Exposure	Duration	Target lesions in lung (LD×SD, millimeter)	Target lesions in brain (LD×SD, millimeter)
2015.04	Baseline	N/A	35 × 24	6.5 × 4.2
2015.06	AP	2 cycles	48 × 42	5.1 × 4.0
2015.07	Icotinib	1 month	22 × 17	2.3 × 2.1
2015.09	Icotinib	3 months	21 × 18	2.0 × 1.8
2015.12	Icotinib	6 months	23 × 21	0
2016.02	Icotinib	8 months	29 × 26	0
2016.04	AZD9291	1 month	17 × 15	N/A
2016.06	AZD9291	3 months	18 × 17	N/A
2016.09	AZD9291	6 months	20 × 16	N/A
2017.01	AZD9291	10 months	61 × 48	0
2017.03	Docetaxel	2 cycles	88 × 39	Multiple lesions
2017.05	Icotinib	2 months	90 × 49	0
2017.06	Icotinib	3 months	64 × 28	N/A
2017.09	Icotinib	6 months	71 × 38	7.1 × 4.2, 3.4 × 2.2
2017.11	Icotinib	8 months	78 × 53	9.5 × 7.1, 6.0 × 5.3
2017.12	WBRT	15F	N/A	N/A
2018.02	AZD9291	1 month	58 × 43	3.0 × 2.1, 0

Abbreviations: *LD* Longest Diameter, *SD* Shortest Diameter, *N/A* Not/Applicable, *AP* Pemetrexed + Cisplatin, *WBRT* Whole Brain Radiation Therapy

## Discussion and conclusions

EGFR-TKI has been emerged as the standard choice for NSCLC patients with EGFR sensitive mutations, while resistant to which appears to be inevitable [2]. EGFR C797S is the most common resistance mechanism in patients who failed to AZD9291 treatment [3]. However, in present case, Leu792H mutation, isoleucine mutations to histidine, was detected rather than C797S after resistant to AZD9291. After repeating icotinib administration, Leu792H mutation vanished again with tumor lesions shrunk, as well as symptoms relieved greatly, which revealed that Leu792H mutation might be another potential mutation account for the resistance to AZD9291. EGFR Leu792H mutation has been reported in a case study containing 3 patients, which suggested that Leu792 mutations could interrupt the binding of AZD9291 to EGFR and potentially cause drug resistance [4]. However, Leu792H mutations in the three patients were accompanied with C797S mutation entirely, and without any further treatment reported. We therefore inferred from the present case that the mutation of EGFR Leu792H is possibly one of the important mechanisms leading to the resistance to AZD9291, whether reversed by repeating icotinib remains uncertain, which, however, could be stand as one of the potential assumption. We will continue to follow up the efficacy and therapeutic duration of the patient, and investigate the mechanism in the re-work of repeating EGFR TKIs.

## Abbreviations

AKT1: RAC-alpha serine/threonine-protein kinase
ALK: Anaplastic lymphoma kinase
ARMS: Amplification refractory mutation system
BRAF: V-raf murine sarcoma viral oncogene homologB1
CA72–4: Carbohydrate antigen 72–4
CEA: Carcino-embryonic antigen
CR: Complete response
CT: Computed tomography
DDR2: Discoidin domain receptor tyrosine kinase 2
EGFR-TKIs: Epidermal growth factor receptor-tyrosine kinase inhibitors
ERBB2: Receptor tyrosine-protein kinase erbB-2
FGFR1: Fibroblast growth factor receptor 1
KRAS: V-Ki-ras2 Kirsten rat sarcoma viral oncogene homolog
MAP2K1: Mitogen-activated protein kinase 1
MET: MNNG HOS transforming gene
MRI: Magnetic resonance imaging
NGS: Next-generation sequencing
NRAS: Neuroblastoma RAS viral oncogene homolog
NSCLC: Non-small cell lung cancer
PD: Progressive disease
PIK3CA: Phosphatidylinosito-4,5-bisphosphate 3-kinase
PNLB: Percutaneous lung biopsy
PR: Partial response
PTEN: Phosphatase and tensin homolog
RECIST: Response evaluation criteria in solid tumors
RET: RET Proto-oncogene
ROS1: ROS proto-oncogene 1
WBRT: Whole brain radiotherapy
